# The invasive stoloniferous clonal plant *Alternanthera philoxeroides* outperforms its co-occurring non-invasive functional counterparts in heterogeneous soil environments – invasion implications

**DOI:** 10.1038/srep38036

**Published:** 2016-11-29

**Authors:** Tong Wang, Jiangtao Hu, Linlin Miao, Dan Yu, Chunhua Liu

**Affiliations:** 1 The National Field Station of Liangzi Lake Ecosystem, Department of Ecology, College of Life Sciences, Wuhan University, Wuhan, 430072, China

## Abstract

Environmental heterogeneity is considered to play a defining role in promoting invasion success, and it favours clonal plants. Although clonality has been demonstrated to be correlated with the invasion success of several species of clonal invasive plants in heterogeneous environments, little is known about how the spatial scale of heterogeneity affects their performance. In addition, the factors that distinguish invasive from non-invasive clonal species and that enhance the invasive potential of clonal exotic invaders in heterogeneous environments remain unclear. In this study, we compared several traits of a noxious clonal invasive species, *Alternanthera philoxeroides*, with its co-occurring non-invasive functional counterparts, the native congener *Alternanthera sessilis*, the exotic *Myriophyllum aquaticum* and the native *Jussiaea repens*, in three manipulative substrates with different soil distribution patterns. We found that the invasive performance of *A. philoxeroides* was not enhanced by heterogeneity and that it was generally scale independent. However, *A. philoxeroides* showed some advantages over the three non-invasives with respect to trait values and phenotypic variation. These advantages may enhance the competitive capacity of *A. philoxeroides* and thus promote its invasion success in heterogeneous environments.

Environmental heterogeneity plays a significant role in biological invasion^1^,^2^,^3^,^4^,^5^. The capacity of clonal growth/production has been considered as a pivotal attribute of exotic invasive clonal plants when facing environmental heterogeneity^6^,^7^,^8^. Clonal stoloniferous plants benefit from environmental heterogeneity because clonal traits (e.g., clonal integration and spatial division of labour) enhance resource exploitation, nutrient exchange and risk spread^9^,^10^,^11^. Hence, environmental heterogeneity favours clonal growth^12^. However, the optimality of clonal growth in heterogeneous environments is scale dependent^13^. For instance, a negative correlation between clonal plant performance and patch size has been demonstrated by several studies^14^,^15^. Moreover, the “environmental heterogeneity hypothesis of invasions” suggests that environmental heterogeneity can promote the invasion success of exotic species^16^. Thus, environmental heterogeneity is likely to promote the performance of invasive clonal plants. Moreover, the performance of invasive clonal plants in heterogeneous environments may be correlated with the spatial scale of heterogeneity.

Trait comparison of co-occurring invasive and non-invasive species to reveal the competitive advantages of invasives over non-invasives generally focuses on two aspects related to invasion mechanisms – trait values and trait plasticity^17^,^18^. A competitive advantage of invasives in trait values refers to advantageous novel traits or extreme trait values of invasive plant species in comparison with non-invasives^19^. Some studies have proposed that invasive plant species display higher values of specific leaf area (SLA), relative growth rate (RGR), body size and belowground traits such as root mass ratio (RMR) and R/S ratio compared with their co-occurring non-invasive species^19^,^20^,^21^. In addition to exhibiting these common traits, stoloniferous clonal plants possess a unique trait of clonality. Invasive clonal plants likely possess a stronger potential of clonality (e.g., clonal integration, spatial division of labour) than do co-occurring, non-invasive clonal species^8^. This stronger potential is expected to facilitate the integration of unevenly distributed resources by invasive clonal plants in heterogeneous environments and thus enhance their performance^10^,^13^,^14^,^15^,^22^. These trait value advantages may promote the rapid colonization and efficient establishment of invasive species in novel and heterogeneous environments.

Trait plasticity refers to the property of phenotypic variation of a genotype in variable environments^23^. Empirical studies have indicated that phenotypic plasticity plays a significant role in biological invasions^17^,^24^,^25^,^26^,^27^,^28^. Adaptive plasticity enables species to extend ecological niche breadth as plastic responses promote advantageous trait expressions in different environments^23^,^29^,^30^. Greater plasticity of invasive species over non-invasive species may help explain the invasion success of exotic invaders in changing environments. However, if disadvantageous phenotypes are induced by plasticity, such plasticity may be detrimental to population fitness^31^,^32^.

In this study, we investigated the relationship between clonal plant invasiveness and environmental heterogeneity in a manipulative experiment. The correlation between clonal performance and the spatial distribution of soil was studied in a stoloniferous weed species that is a detrimental invasive in many regions worldwide, – *Alternanthera philoxeroides*. Three non-invasive wetland stoloniferous clonal plant species that co-occur with *A. philoxeroides* – a native congener *Alternanthera sessilis*, an exotic non-invasive *Myriophyllum aquaticum* and the native *Jussiaea repens* – were selected as comparison species to explore the invasion mechanism of *A. philoxeroides* in heterogeneous environments. *Alternanthera philoxeroides* competes with the three non-invasive counterparts as these four species occupy similar ecological niche in the field (personal observation). The following hypotheses were postulated: i. *A. philoxeroides* benefits from soil heterogeneity, and the benefit strength is patch-scale dependent. ii. Compared with the three non-invasive functional counterparts, the invasive *A. philoxeroides* displays some trait advantages in the presence of different soil distribution patterns.

## Materials and Methods

### Plant materials

#### Alternanthera philoxeroides (Martius) Grisebach

*Alternanthera philoxeroides* is a stoloniferous clonal herb with a strong dispersal ability in terrestrial, semi-aquatic and aquatic habitats^33^. This species commonly reproduces via regeneration from clonal fragments in China. Previous studies have found that phenotypic plasticity in its clonal characteristics rather than genetic differentiation helps explain its successful invasion in a wide range of habitats in China^25^,^34^,^35^.

#### Alternanthera sessilis (L.) R. Br

As a stoloniferous clonal herb with sexual reproduction, *A. sessilis* is mainly distributed in moist habitats such as swamps, wetlands and the edges of ditches and canals^36^.

#### Myriophyllum aquaticum (Vell.) Verdcourt

Originating from South America as *A. philoxeroides*, *M. aquaticum* survives in both aquatic and semi-aquatic habitats^37^. Asexual clonal propagation is its major reproductive mode in China^38^.

#### Jussiaea repens L

*Jussiaea repens* is a dominant native species occurring in sub-tropical and tropical regions in China^39^. Its capacity for forming dense mats makes it a noxious “channel blocker” in Europe^40^. Its strong stoloniferous clonality also facilitates its dispersal in moist environments.

### Experimental design

On June 25, 2015, fifty clonal ramets sharing similar morphology (10-cm-long shoots with 6 leaves for *A. philoxeroides*, *A. sessilis* and *J. repens* and 10-cm-long shoots with approximately 14 leaf whorls for *M. aquaticum*) were collected of each species from mono-populations of each species in the riparian zone of Liangzi Lake (30°05′–30°18′N, 114°21′–114°39′E). All of the collected plant materials were pre-cultivated in sandy clay three times for approximately two months before the experiment set-up. Twenty-four morphologically identical plants without branches were then selected of each species. Six plants of each species were randomly selected and dried to determine the initial biomass. The eighteen remaining plants (height: approximately 15 cm; initial biomass: mean ± SE, 0.3042 ± 0.0112 g for *A. philoxeroides*, 0.3778 ± 0.0124 g for *A. sessilis*, 0.3603 ± 0.0127 g for *M. aquaticum* and 0.4652 ± 0.0193 g for *J. repens*) were selected for the experiment.

**Figure 1:**
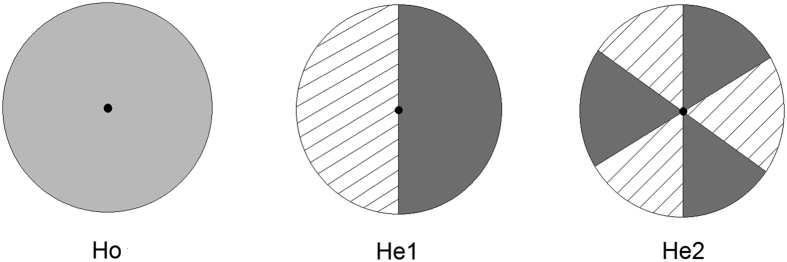
Schematic representation of the three substrate types with different soil distribution patterns. Area in light grey represents mixture of same volume of clay and sand in Ho. Area in dark grey represents clay patch in He1 and He2. Area with slashes represents sand patch in He1 and He2. Central black points in Ho, He1 and He2 represent where the plant were cultivated during the experiment set-up

Seventy-two round basins (diameter: 45 cm, height: 45 cm) were selected as mesocosms. Three substrate types were designed: (1) homogeneous substrate (Ho), comprised of a homogenous mixture of equal volumes of clay (mean ± SE, five replicates, 0.055 ± 0.0063 g.g^−1^ organic matter; particle size: <75 μm) and sand (mean ± SE, five replicates, 0.004 ± 0.0004 g.g^−1^ organic matter, particle size: 330–880 μm); (2) heterogeneous substrate, composed of two contrasting patches of equal volumes of clay and sand (He1); and (3) heterogeneous substrate with six adjoining patches of equal volumes of clay and sand (He2) (Fig. 1). All of the substrates were 15 cm in thickness. The total amount of each resource type was identical across substrate types. All of the selected seedlings were cultivated into the centre of each substrate. Each treatment was replicated 6 times. All of the treatments and replicates were randomly positioned on an outdoor cement platform (10 mL × 10 mW). To produce a moist habitat, 1-cm-deep lake water (N:P = 0.71:0.04 mg.L^−1^) was maintained above the substrate surface throughout the experimental period. The experiment was established on August 30, 2015.

### Harvest and measurement

All of the plant materials were harvested on November 25, 2015, after 87 days of growth. Immediately after harvest, stolon length was measured and ramet number was counted for each replicate. Five leaves of each replicate on the 3^rd^ to 5^th^ leaf node on the apical end of the stolon were selected to gauge leaf area (Li-3100 Area Meter, Li. Cor. Inc., Lincoln, Nebraska, USA) except for the leaves of *M. aquaticum*. As the leaves of *M. aquaticum* are tiny and needle-like, leaf area could not be precisely measured by the leaf area metre. Therefore, five leaves on the 5^th^ to 7^th^ leaf node on the apical end of the stolon were randomly selected and scanned (Epson V850 Perfection Pro, Seiko Epson Corp., Japan) to produce 1:1 high definition images (tiff format, 600 dpi) on a whiteboard with a ruler. Then, ImageJ 1.46 (National Institute of Health, Bethesda, Maryland, USA) was used to analyse the leaf area. The selected leaves of the four species were oven dried at 70 °C for 72 h to determine dry biomass. Specific leaf area (SLA) was calculated as follows^41^:


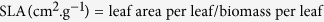


The mean SLA values of the selected five leaves of each replicate were used for data analysis.

The remnant parts of the plant materials were separated into leaves, stolon and roots (leaves, stolon, flowers and roots for *A. sessilis*) for each replicate. The biomass of each plant part in each replicate was determined after oven drying at 70 °C for 72 h. Total biomass, relative growth rate (RGR), leaf mass ratio (LMR), stolon mass ratio (SMR), root mass ratio (RMR) and root-shoot ratio (R/S ratio) were calculated as follows^41^:


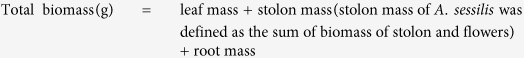



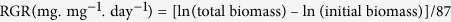



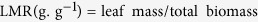



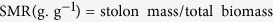



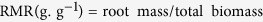



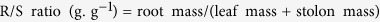


### Statistical analysis

All of the data met the assumptions of normality and homogeneity of variance prior to analysis. Stolon length, ramet number and total biomass were each transformed using the functions of log(log(x)), log(x) and log(x + 1), respectively. RGR, SMR and RMR were transformed using the square root(x) function. R/S ratio was transformed using the x^1/4^ function. Two-way ANOVA was implemented to test for the effects of species and substrate type on plant traits. If a significant treatment effect was detected, post hoc pair-wise comparisons of means were performed to examine differences between treatments using Duncan’s test for multiple comparisons. Dunnett’s test was used to examine the trait value differences between *A. philoxeroides* and each of the three non-invasive species. All of the analyses were performed using SPSS 22.0 (SPSS, Chicago, Illinois, USA).

## Results

The species exerted significant effects on all traits, and the substrate type exerted significant effects on all traits except SLA (Table 1). Significant interactive effects of species and substrate type were observed on all traits except SLA, RMR and R/S ratio (Table 1).

### Phenotypic variation

**Figure 2:**
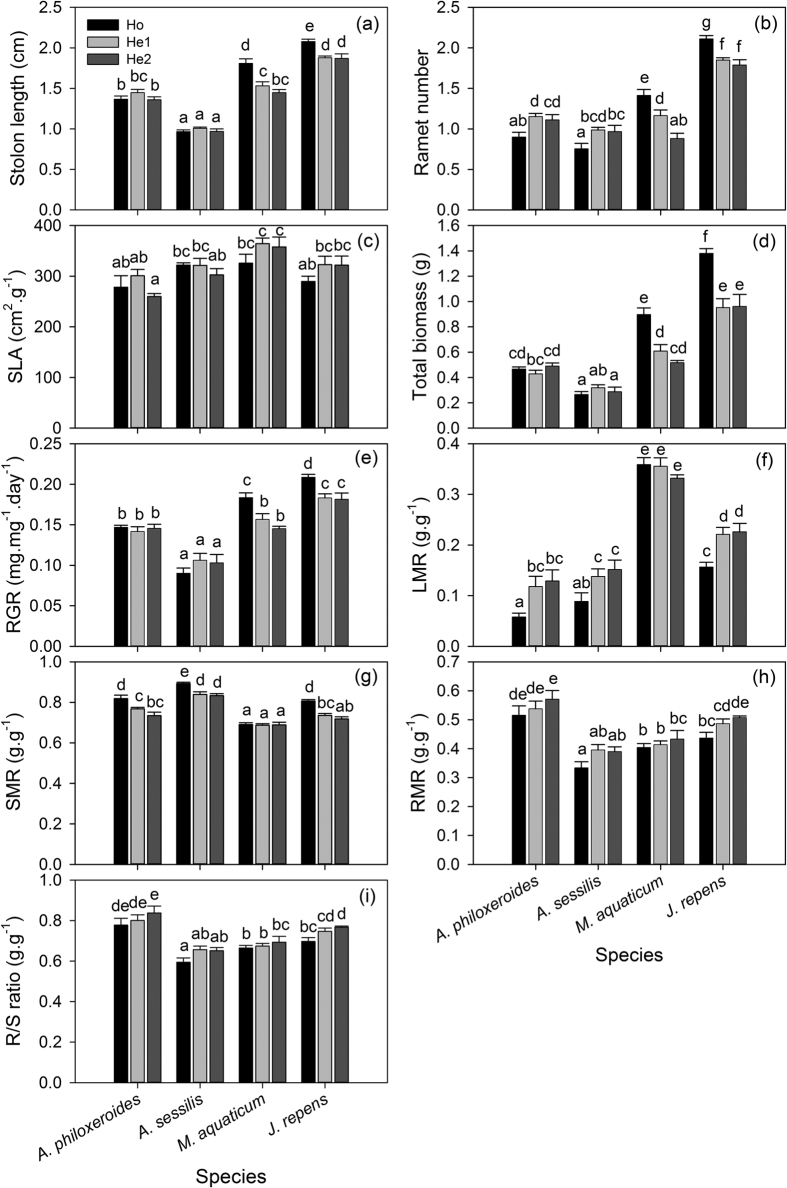
Trait value variation of the four species in different substrates. Values represent mean ± SE. Vertical bars with different letters represent significant differences (*P* < 0.05).

*Alternanthera philoxeroides* showed similar trait values of stolon length, SLA, total biomass, RGR, RMR and R/S ratio among the different substrates (Fig. 2a,c,d,e,h,i). Ramet number and LMR were 25.9% and 113.5% (significantly) higher and SMR was 8.2% (significantly) lower in the heterogeneous substrates than in Ho (Fig. 2b,f,g). No significant differences in ramet number, LMR and SMR of *A. philoxeroides* were shown between the He1 and He2 treatments (Fig. 2b,f,g). *Alternanthera sessilis* showed approximately the same responses as *A. philoxeroides* of all measured traits in the Ho, He1 and He2 treatments (Fig. 2).

*Myriophyllum aquaticum* showed 17.7% and 17.8% (significantly) lower values of stolon length and RGR, respectively, in the heterogeneous substrates than in Ho (Fig. 2a,e). Decreases of 37.6% and 42.4% in ramet number and total biomass, respectively, were observed between the Ho and He2 treatments (Fig. 2b,d). Similar values of SLA, biomass allocation and R/S ratio were observed among all substrates (Fig. 2c,f–i).

*Jussiaea repens* showed 9.8%, 13.8%, 30.7%, 12.6% and 9.9% (significantly) lower values of stolon length, ramet number, total biomass, RGR and SMR, respectively, in heterogeneous substrates than in Ho (Fig. 2a,b,d,e,g). SLA was homogeneous across all substrates (Fig. 2c). LMR was significantly higher by 42.8% in heterogeneous substrates than in Ho (Fig. 2f). Increases of 16.1% and 10.1% in RMR and R/S ratio, respectively, were shown in the He2 treatment relative to the Ho treatment, with significant differences observed between Ho and He2 (Fig. 2h,i).

### Trait values

*Alternanthera philoxeroides* showed 41.8%, 58.9%, 44.9%, 45.1% and 27.0% (significantly) higher values of stolon length (*P* < 0.001), total biomass (*P* = 0.010), RGR (*P* < 0.001), RMR (*P* < 0.001) and R/S ratio (*P* < 0.001), respectively, than *A. sessilis* on average (Fig. 2a,d,e,h,i). Similar values of ramet number (*P* = 0.078) and LMR (*P* = 0.249) were shared between *A. philoxeroides* and *A. sessilis* on average (Fig. 2b,f). *Alternanthera philoxeroides* showed 11.3% and 9.52% (significantly) lower values of SLA (*P* = 0.014) and SMR (*P* < 0.001), respectively, than *A. sessilis* on average (Fig. 2c,g).

*Alternanthera philoxeroides* showed 12.8%, 19.9%, 31.5%, 10.5% and 70.9% (significantly) lower values of stolon length (*P* < 0.001), SLA (*P* < 0.001), total biomass (*P* = 0.001), RGR (*P* = 0.017) and LMR (*P* < 0.001), respectively, than *M. aquaticum* on average (Fig. 2a,c–f). Similar values of ramet number were shared between *A. philoxeroides* and *M. aquaticum* on average (*P* = 0.349) (Fig. 2b). *Alternanthera philoxeroides* showed 12.3%, 29.9% and 18.9% (significantly) higher values of SMR (*P* < 0.001), RMR (*P* < 0.001) and R/S ratio (*P* < 0.001), respectively, than *M. aquaticum* on average (Fig. 2g–i).

*Alternanthera philoxeroides* showed 28.4%, 45.0%, 10.2%, 57.9%, 24.2% and 49.6% (significantly) lower values of stolon length (*P* < 0.001), ramet number (*P* < 0.001), SLA (*P* = 0.033), total biomass (*P* < 0.001), RGR (*P* < 0.001) and LMR (*P* < 0.001), respectively, than *J. repens* on average (Fig. 2a–f). Similar values of SMR were shared between *A. philoxeroides* and *J. repens* on average (*P* = 0.299) (Fig. 2g). *Alternanthera philoxeroides* showed 13.5% and 9.2% (significantly) higher values of RMR (*P* = 0.002) and R/S ratio (*P* = 0.002), respectively, than *J. repens* on average (Fig. 2h,i).

## Discussion

### Soil heterogeneity may not be an optimal promoter of invasive performance in *A. philoxeroides*

Environmental heterogeneity is considered to favour clonal growth because stoloniferous clonal plants can display a variety of clonal functional traits that allow them to cope with environmental heterogeneity^42^. Clonal functional traits such as foraging behaviour, clonal integration and spatial division of labour are expected to benefit the performance of clonal plants in heterogeneous environments because they facilitate the exploitation of benign resources, internal exchange of resources and spread of risk^9^,^10^,^11^. Wijesinghe and Hutchings^14^,^15^ found a negative correlation between the performance (biomass) of the stoloniferous herb *Glechoma hederacea* and patch size. Moreover, Zhou *et al*.^43^ suggested that heterogeneity might be a significant driving factor of clonal plant invasion if a strong, positive response to fine-scale nutrient heterogeneity is common. In our study, based on our trait measurements, we found that *A. philoxeroides* gained some benefits from soil heterogeneity regardless of patch size. First, *A. philoxeroides* recruited more ramets in heterogeneous substrates than in the homogeneous substrate (Fig. 2b). As a low efficiency of sexual reproduction tends to exist in clonal plants^44^, clonal growth/reproduction is likely an important proxy of fitness in clonal plants^45^. A stronger recruitment of ramets may represent stronger propagule pressure related to the invasiveness potential of clonal invasive plants^45^. Second, a higher LMR and a lower SMR were simultaneously shown in heterogeneous environments relative to the homogeneous environment (Fig. 2f,g). This trade-off of biomass allocation might enable *A. philoxeroides* to invest more energy in light utilization rather than in the support structures in heterogeneous environments. The promotion of photosynthetic capacity might be induced by a “leafier” modular system^46^. These benefits derived from heterogeneity may confer *A. philoxeroides* with adaptive advantages under environmental heterogeneity.

However, our study found that *A. philoxeroides* displayed generally similar, scale-independent performance in most traits under different soil distribution patterns (Fig. 2a,c,d,e,h,i). A potential explanation for this result is that spatial division of labour, involving the integration of inter-connected clonal modules of *A. philoxeroides*, might help to effectively access the heterogeneous distribution of soil resources and buffer the soil heterogeneity^10^,^22^,^47^,^48^,^49^. Moreover, a long-term experimental study by Fransen and de Kroon^50^ showed that the extent to which environmental heterogeneity favours clonal plants weakens over time. Recently, Dong *et al*.^51^ showed that the performance of *A. philoxeroides* can be enhanced by clonal integration in homogeneous environments. Thus, the homogenization of plant performance in *A. philoxeroides* might be expected in heterogeneous environments. We predict that the benefit of environmental heterogeneity to clonal plants may be correlated with temporal scale.

In contrast to previous empirical findings^14^,^15^, *A. philoxeroides* benefited less from soil heterogeneity, and its performance was not patch-scale dependent. Environmental heterogeneity is unlikely to be a primary promoter of invasive success in *A. philoxeroides*.

### Some advantageous traits of *A. philoxeroides* relative to those of its non-invasive functional counterparts contribute to its invasion success

An important invasion mechanism of successful invasive species is the ability of invasive species to outperform co-occurring non-invasive species in trait values and/or trait plasticity^19^,^21^,^27^,^28^,^52^. Advantageous trait values enable invasive species to outcompete non-invasive species and thus facilitate the establishment of invasive species in recipient habitats^8^,^19^. Adaptive trait plasticity promotes the optimal trait expression of invasive species in changing environments and thus enhances the ecological amplitude of invasive species when encountering a broad range of habitats^23^,^25^,^29^,^30^. In our study, we found that invasive *A. philoxeroides* possessed some advantages with respect to trait values and phenotypic variation compared with its non-invasive functional counterparts.

In terms of trait values, first, *A. philoxeroides* generally showed significantly higher trait values of stolon length, total biomass and RGR and significantly lower trait values of SMR in comparison with *A. sessilis* (Fig. 2a,d,e,g). As a proxy for habitat exploitation, stolon elongation is positively correlated with the capacity for dispersal and occupation in stoloniferous clonal plants^53^,^54^. The cost of stolon elongation for *A. philoxeroides* was lower than that for *A. sessilis* as *A. philoxeroides* invested less biomass into stolon elongation than did *A. sessilis*. A higher benefit/cost ratio may enable *A. philoxeroides* to rapidly colonize when competing with *A. sessilis*. Proxies for fitness commonly depend on biomass, size or growth rate^19^,^55^. Hence, higher biomass accumulation may reflect higher fitness for *A. philoxeroides*. Previous studies have suggested that RGR profoundly influences the competitive ability and recruitment of exotic invaders^19^,^20^,^56^. In the early phase of the invasion process, a higher RGR is likely an indicator of the more rapid establishment of exotic invaders in foreign habitats^57^. Second, *A. philoxeroides* generally showed significantly higher biomass allocation to roots than did the three non-invasive species (Fig. 2h,i). This higher root mass allocation may help *A. philoxeroides* to utilize soil nutrients more efficiently and/or enhance nutrient storage for asexual regeneration as storage roots are propagative organs in *A. philoxeroides*^58^,^59^. Previous studies have also demonstrated that successful invasive species likely benefit more from biomass allocation to belowground parts than do co-occurring non-invasive species, especially in infertile habitats^19^,^52^,^57^,^60^.

Overall, *A. philoxeroides* displayed significantly lower values of stolon length, SLA, total biomass, RGR and LMR than did *M. aquaticum*, and significantly lower values of stolon length, ramet number, SLA, total biomass, RGR and LMR than did *J. repens* on average (Fig. 2a–f). In terms of trait values, *A. philoxeroides* showed absolute inferiority in overall plant performance related to photosynthetic capacity and vegetative growth compared with *M. aquaticum* and *J. repens*^19^,^20^. However, *A. philoxeroides* displayed stronger adaptation to heterogeneity than did *M. aquaticum* and *J. repens* with respect to phenotypic variation. First, *A. philoxeroides* maintained approximately consistent values of stolon length, total biomass and RGR in all substrates and recruited more ramets in heterogeneous substrates than in the homogeneous one. In contrast, *M. aquaticum* and *J. repens* showed lower values of these traits under heterogeneity (Fig. 2a,b,d,e). Second, *A. philoxeroides* increased leaf mass allocation and decreased stolon mass allocation under heterogeneity, whereas *M. aquaticum* showed approximately identical biomass allocation in different substrates (Fig. 2f,g). Inconsistent with the hypothesis that environmental heterogeneity favours clonal plants over non-clonal ones^12^,^61^, both of the non-invasive clonal species *M. aquaticum* and *J. repens* showed unfavourable responses to environmental heterogeneity, whereas *A. philoxeroides* showed generally consistent performance across homogeneous and heterogeneous substrates. Our results are in partial agreement with a previous study by You *et al*.^8^ that found stronger clonal integration in *A. philoxeroides* than in *J. repens* in experimentally manipulated heterogeneous environments. Stronger clonality (e.g., clonal integration and spatial division of labour) in *A. philoxeroides* than in *M. aquaticum* and *J. repens* likely enabled *A. philoxeroides* to integrate the heterogeneity more efficiently and thus enhance its invasive potential. In contrast, the maladaptive trait plasticity shown by *M. aquaticum* and *J. repens* may confer fitness costs from an evolutionary perspective^31^,^32^.

In summary, soil heterogeneity is unlikely to be a primary promoter of invasive success in *A. philoxeroides*. This conclusion is based on our finding of approximately consistent performance maintenance across different soil distribution patterns. However, some advantages of *A. philoxeroides* over its non-invasive co-occurring functional counterparts with respect to trait values and phenotypic variation may help explain the successful invasion of the noxious clonal weed *A. philoxeroides* in heterogeneous environments. Additionally, based on our observations, we predict that soil texture, temporal scale and growing season are likely to influence the growth of the four evaluated species. Future studies should focus on how diversified environmental heterogeneity due to various ecological factors affects the invasive performance of clonal alien species.

## Additional Information

**How to cite this article**: Wang, T. *et al*. The invasive stoloniferous clonal plant *Alternanthera philoxeroides* outperforms its co-occurring non-invasive functional counterparts in heterogeneous soil environments - invasion implications. *Sci. Rep.* **6**, 38036; doi: 10.1038/srep38036 (2016).

**Publisher's note:** Springer Nature remains neutral with regard to jurisdictional claims in published maps and institutional affiliations.

## Figures and Tables

**Table 1:** *F*-value and significances of two-way ANOVA of the effects of species and substrate type on the growth traits of the four species.

	Species (S)		Substrate type (T)		S × T	
	*F*	*P*	*F*	*P*	*F*	*P*
**Stolon length**	322.4755	**<0.001**	13.943	**<0.001**	7.004	**<0.001**
**Ramet number**	175.140	**<0.001**	4.139	**0.021**	11.492	**<0.001**
**SLA**	11.227	**<0.001**	2.676	0.077	1.137	0.352
**Total biomass**	169.683	**<0.001**	20.587	**<0.001**	8.812	**<0.001**
**RGR**	111.105	**<0.001**	5.056	**0.009**	4.219	**0.001**
**LMR**	154.778	**<0.001**	10.478	**<0.001**	2.438	**0.036**
**SMR**	110.464	**<0.001**	29.362	**<0.001**	3.541	**0.005**
**RMR**	34.653	**<0.001**	6.275	**0.003**	0.406	0.872
**R/S ratio**	33.601	**<0.001**	6.074	**0.004**	0.400	0.876
